# The Synergistic Effect of Proanthocyanidin and HDAC Inhibitor Inhibit Breast Cancer Cell Growth and Promote Apoptosis

**DOI:** 10.3390/ijms241310476

**Published:** 2023-06-22

**Authors:** Tsz Ki Wang, Shaoting Xu, Yuanjian Fan, Jing Wu, Zilin Wang, Yue Chen, Yunjian Zhang

**Affiliations:** 1 Department of Breast Surgery, The First Affiliated Hospital of Sun-Yat Sen University, Guangzhou 510080, China; wangtszki@mail2.sysu.edu.cn (T.K.W.);; 2 Laboratory of Surgery, The First Affiliated Hospital of Sun-Yat Sen University, Guangzhou 510080, China; 3 Department of Thyroid Surgery, The First Affiliated Hospital of Sun-Yat Sen University, Guangzhou 510080, China

**Keywords:** histone deacetylase inhibitor, breast cancer, proanthocyanidins, synergistic effect, cell proliferation, apoptosis, steroid biosynthesis, extracellular matrix receptor pathway

## Abstract

Histone deacetylase inhibitor (HDACi) is a drug mainly used to treat hematological tumors and breast cancer, but its inhibitory effect on breast cancer falls short of expectations. Grape seed proanthocyanidin extract (GSPE) with abundant proanthocyanidins (PAs) has been explored for its inhibition of HDAC activity in vitro and in vivo. To enhance HDACi’s effectiveness, we investigated the potential of PA to synergistically enhance HDACi chidamide (Chi), and determined the underlying mechanism. We evaluated the half-inhibitory concentration (IC50) of PA and Chi using the cell counting kit 8 (CCK8), and analyzed drugs’ synergistic effect with fixed-ratio combination using the software Compusyn. Breast cancer cell’s phenotypes, including short-term and long-term proliferation, migration, invasion, apoptosis, and reactive oxygen species (ROS) levels, were assessed via CCK8, clone-formation assay, wound-healing test, Transwell Matrigel invasion assay, and flow-cytometry. Protein–protein interaction analysis (PPI) and KEGG pathway analysis were used to determine the underlying mechanism of synergy. PA + Chi synergistically inhibited cell growth in T47D and MDA-MB-231 breast cancer cell lines. Short-term and long-term proliferation were significantly inhibited, while cell apoptosis was promoted. Ten signaling pathways were identified to account for the synergistic effect after RNA sequencing. Their synergism may be closely related to the steroid biosynthesis and extracellular matrix (ECM) receptor interaction pathways. PA + Chi can synergistically inhibit breast cancer cell growth and proliferation, and promote apoptosis. These effects may be related to steroid biosynthesis or the ECM receptor pathway.

## 1. Introduction

Breast cancer is the most common cancer among women worldwide, with over 2.26 million reported cases out of a total of 9.23 million female cancer cases. Chinese women account for about 18.5% of these cases [1]. Approximately 70% of breast cancer cases are estrogen-receptor-positive (ER+), but many patients develop primary or secondary resistance to hormone therapy [2,3].

Histone deacetylase (HDAC) inhibitors are adjunctive drugs that can help cancer cells regain sensitivity to hormone therapy [4]. HDAC is an enzyme that regulates histone lysine residue acetylation, thereby altering gene expressions by modifying the spatial structure of DNA [5,6,7]. In malignancies, tumor-suppressor genes were transcription inhibited and resulted in tumor progression [8]. HDAC inhibitors (HDACi) have been used to treat late-stage breast cancer, but their effectiveness varies among patients, highlighting the importance of identifying the responders to this treatment [9,10,11,12,13]. Therefore, investigating the mechanism by which HDACi affect breast cancer cells is of significant importance. Chidamide (or named tucidinostat, CS055; Chi) is a novel synthetic benzamide-type selective HDACi developed in China that targets type I and IIa HDACs (HDAC1, HDAC2, HDAC3 and HDAC10). Although it is included in the second-line treatment regimen in the Chinese Society of Clinical Oncology (CSCO) guidelines based on successful results from the ACE study, the clinical effectiveness of HDACi in solid tumors has been limited [14,15]. Therefore, exploring methods to enhance the efficacy of HDACi is warranted.

Proanthocyanidins (PAs) are flavonoids that are widely found in fruits, plant peels, seeds, and processed beverages, such as green tea, black tea, and grape seed extract (GSE/GSPE) [16,17,18,19,20,21,22]. PAs have been shown to have multiple therapeutic effects in both malignant and normal tissues, including anti-angiogenesis, pro-apoptosis, and epigenetic changes. Combining PAs with other drugs can also enhance the effects of the other drugs [23,24]. Previous studies have reported that GSPE regulates histone acetylation and decreases HDAC activity in both in vivo and in vitro settings, whether used individually or in combination [23,25]. Therefore, the use of a safe and easily accessible PA as an HDACi sensitizer to promote the effect of the HDACi could hold significant promise for patients with advanced breast cancer.

This study aimed to investigate the synergistic inhibitory effect of the combined administration of PA and Chi in the treatment of breast cancer. In addition, we aimed to identify potential targets or pathways associated with the synergistic anti-tumor growth and development effects of this combination therapy through transcriptome sequencing and bioinformatic analysis. The findings could provide a new therapeutic direction for HDACi as an adjuvant drug in treating advanced breast cancer.

## 2. Results

### 2.1. Proanthocyanidin in Combination with an HDACi Exerts Synergistic Anti-Tumor Effects on Breast Cancer Cell Lines

(a) Breast cancer cell line selection based on HDAC expression screening from a single-cell RNA expression profile

**Figure 1:**
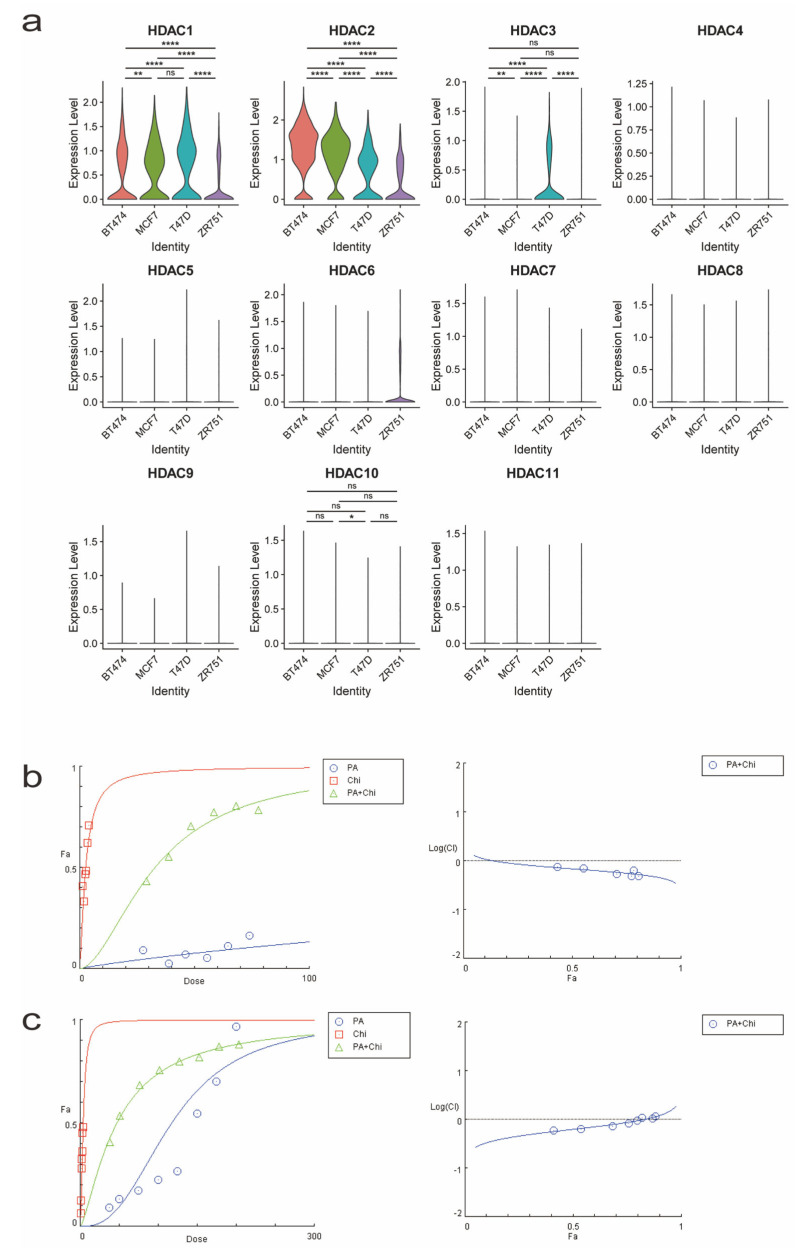
(**a**) Violin plot depicting the expression levels of HDACs in commonly used estrogen-positive breast cancer cell lines (BT474, MCF7, T47D, and ZR751). (For gene expression level, *n* = 2158 (BT474), *n* = 839 (MCF7), *n* = 825 (T47D), and *n* = 943 (ZR751). *^ns^ p* > 0.05, * *p* < 0.05, ** *p* < 0.01, or **** *p* < 0.0001, statistical significances were assessed by one-way ANOVA after the D’Agostino-Pearson normality test). (**b**) Cell inhibition curve and logarithmic combination index (LogCI) value after treating T47D cells with a proanthocyanidin and chidamide (PA + Chi) ratio of 18.5 to 1. LogCI value < 0 indicates synergistic effect between drugs. (**c**) Cell inhibition curve and LogCI value after treating MDA-MB-231 cells with a PA + Chi ratio of 50 to 1.

Chidamide (Chi) is an HDACi primarily used in patients with ER+ breast cancer. To select a suitable cell line for our experiments, we screened a single-cell RNA sequencing profile of four commonly used ER+ breast cancer cell lines (BT474, MCF7, T47D, and ZR751) in the Gene Expression Omnibus (GEO) database. We observed variations in the expression levels of *HDAC1*, *HDAC2*, *HDAC3* and *HDAC10* mRNAs targeted by chidamide among the ER+ breast cancer cell lines. Notably, the T47D cell line exhibited significant expression of *HDAC1* and *HDAC3* genes compared to the other cell lines, as shown in the violin plot (Figure 1a). Therefore, we selected the T47D cell line and the widely accessible triple-negative breast cancer (TNBC) cell line MDA-MB-231 as the primary cell lines for subsequent experiments.

(b) Proanthocyanidins and chidamide synergistically inhibit breast cancer cell growth

To evaluate the inhibitory effects of applied drugs, we first determined the half-inhibitory concentrations (IC50) of grape seed extract powder (the main component is PA) and Chi, separately. The IC50 of PA for the T47D cell line was 90.74 µg/mL, and the IC50 of Chi was 9.778 µM (Figure S1a,b). In MDA-MB-231 cells, the IC50 of PA was 87.9 µg/mL, and that of Chi was 3.096 µM (Figure S1c,d). These findings indicated that both drugs exhibit growth-inhibiting effects on breast cancer cell lines of different molecular types. Based on the IC50 values, we tested various combinations of PA and Chi and analyzed them using a synergistic model. We observed a synergistic inhibitory effect when treating T47D cells with a PA and Chi ratio of 18.5 to 1 (18.5:1) (Figure 1b, Table 1). Similarly, MDA-MB-231 cells showed a synergistic effect at a ratio of 50 to 1 (50:1) (Figure 1c, Table 2).

### 2.2. Analysis of the Cell Function of Proanthocyanidin in the Synergistic HDACi Inhibition of Breast Cancer Cell Growth

(a) Cell proliferation was significantly inhibited by treatment with PA + Chi

**Figure 2:**
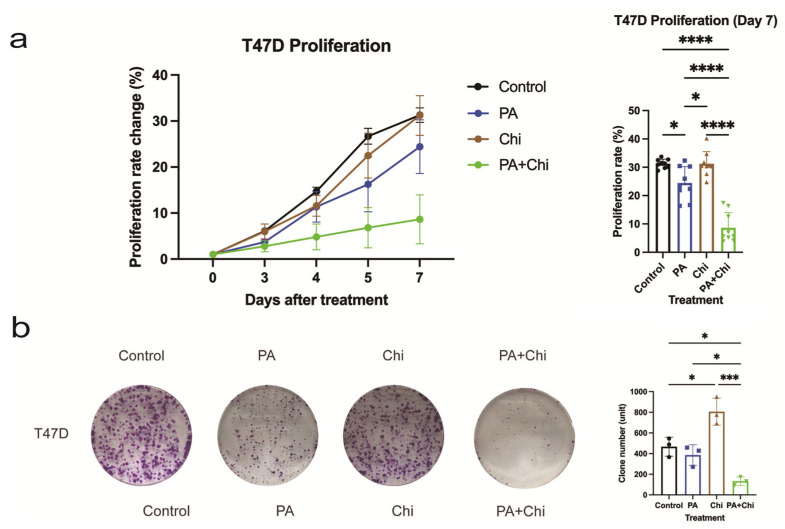

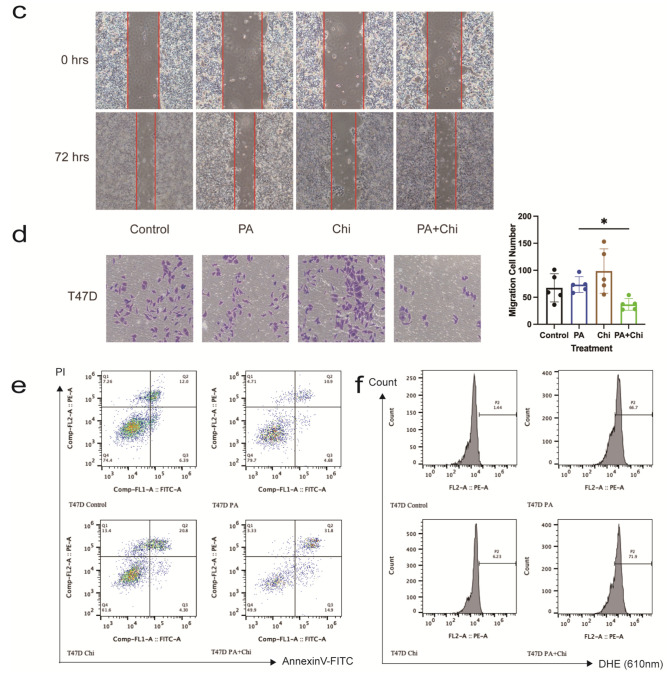
(**a**) Proliferation curves of T47D cells treated with proanthocyanidins (PA), chidamide (Chi), and their combination (PA + Chi), and the difference at day 7 after treatment. (For proliferation rate (%), *n* = 9 (Control), *n* = 9 (PA), *n* = 9 (Chi), and *n* = 9 (PA + Chi). * *p* < 0.05, or **** *p* < 0.0001). (**b**) Clone formation assay showing the number of cell colonies formed by T47D cells treated with PA, Chi, PA + Chi, and the control group. (For clone number present in histogram, *n* = 3 (Control), *n* = 3 (PA), *n* = 3 (Chi), and *n* = 3 (PA + Chi). * *p* < 0.05, or *** *p* < 0.001). (**c**) Wound-healing assay results comparing T47D cell migration from 0 h to 72 h after treatment with PA, Chi, and PA + Chi. (×40) (**d**) Transwell assay evaluating the invasive ability of T47D cells treated with PA, Chi, PA + Chi, and the control group. Representative photographs and quantification of migrated cells through the upper chamber are shown. The number of cells adhering to the outside membrane after 48 h was counted in five randomly selected views (×200; For migration cell number, *n* = 5 (Control), *n* = 5 (PA), *n* = 5 (Chi), and *n* = 5 (PA + Chi). * *p* < 0.05). (**e**) Apoptosis levels of T47D cells treated with PA, Chi, or PA + Chi compared to the control group. Upper left: control; upper right: PA; bottom left: Chi; bottom right: PA + Chi. (**f**) Percentage increase in reactive oxygen species (ROS) in T47D cells treated with PA, Chi, and PA + Chi. No synergistic effect on ROS percentage change was observed. Statistical analysis for all quantitative multi-group comparisons in the histograms was performed using one-way ANOVA (Brown-Forsythe and Welch ANOVA tests for data with non-equal standard deviations).

To investigate the impact of the combination of PA and Chi on breast cancer development, we conducted several tests to assess cancer cells’ phenotypic changes. Initially, we used the CCK8 assay to evaluate the short-term proliferation capacity of T47D cells after monotherapy (PA or Chi) or combination therapy (PA + Chi) at 0, 3, 4, 5, and 7 days. The results showed that cell proliferation rate in the PA + Chi group was lower than in the PA or Chi treatment groups. Furthermore, the differences in cell proliferation among the groups increased in significance with an increase in culture time (*p* < 0.05) (Figure 2a and Figure S2a,b).

(b) Clone-formation was dramatically inhibited by treatment with PA + Chi

The number of cell colonies formed in the PA + Chi group (133 ± 40) was significantly lower than in the control, PA, or Chi group (467 ± 92, 386 ± 100, 806 ± 131, respectively) (all, *p* < 0.05). Moreover, there were statistically significant differences between the control group and PA group, the PA group and Chi group, and the Chi group and PA + Chi group (Figure 2b). These findings suggested that the combination of PA + Chi can effectively reduce the long-term proliferation ability of T47D breast cancer cells.

(c) Wound healing was not different in cells treated with PA, Chi, or PA + Chi

To evaluate cell migration ability, a wound-healing experiment was conducted 72 h after treatment with PA, Chi, and PA + Chi. In the T47D cell line, the results showed no significant difference in cell scratch healing ability within all groups (Figure 2c, *p* > 0.05). This finding suggested that the synergistic effect of PA + Chi does not manifest by altering migration ability.

(d) No significant differences in cancer cell invasion inhibition via PA + Chi

To investigate the impact of PA + Chi on cell invasion ability, we conducted the Transwell Matrigel invasion assay using each drug individually and in combination (PA + Chi). The results demonstrated that compared to treatment with individual drugs, the number of cells invading through the Transwell permeable membrane (containing Matrigel gel) was lower in the PA + Chi group. However, only the difference between the PA group and the PA + Chi group reached statistical significance (*p* < 0.05), while no statistical differences were observed among other groups (*p* > 0.05) (Figure 2d). This finding suggested that the synergistic effect of PA + Chi does not directly inhibit tumor invasion, but may be mediated through the associated suppression of tumor growth.

(e) PA + Chi effectively promotes apoptosis

Flow cytometry was used to access apoptotic changes in T47D cells following treatment with PA, Chi, and PA + Chi. The results revealed no significant difference in the proportion of apoptotic cells between the control group (6.39% early apoptosis; 12.0% late apoptosis) and the PA group (4.68% early apoptosis; 10.9% late apoptosis). Notably, the Chi group exhibited a significant increase in late apoptosis (4.3% early apoptosis; 20.8% late apoptosis), with levels approximately two times that of the control group. The PA + Chi group demonstrated a significant elevation in both early and late stage apoptotic cells (14.9% early apoptosis; 31.8% late apoptosis), collectively accounting for half of all cells analyzed (Figure 2e). These findings conferred that the combination of PA + Chi may enhance cell apoptotic ability.

(f) The level of reactive oxygen species (ROS) Was similar between cells treated with PA and PA + Chi

The ROS detection kit and flow cytometry were employed to analyze the alterations in ROS level in T47D cells following treatment with PA, Chi, and PA + Chi. The results showed that the ROS levels in the control and Chi-treated groups were relatively low (1.44% and 6.23%, respectively), with no significant difference between the two groups. Remarkably, the cell treated with PA and PA + Chi exhibited a significant increase in ROS level (66.7% and 71.9%, respectively) compared to the control group. However, there was no significant difference in ROS levels between the two groups (Figure 2f), indicating that the elevation in ROS was primarily caused by PA, and PA + Chi did not have a synergistic effect on ROS levels.

### 2.3. Differential Gene Expression (DEG) Analysis and Gene Enrichment Analysis of RNA-Sequencing

**Figure 3:**
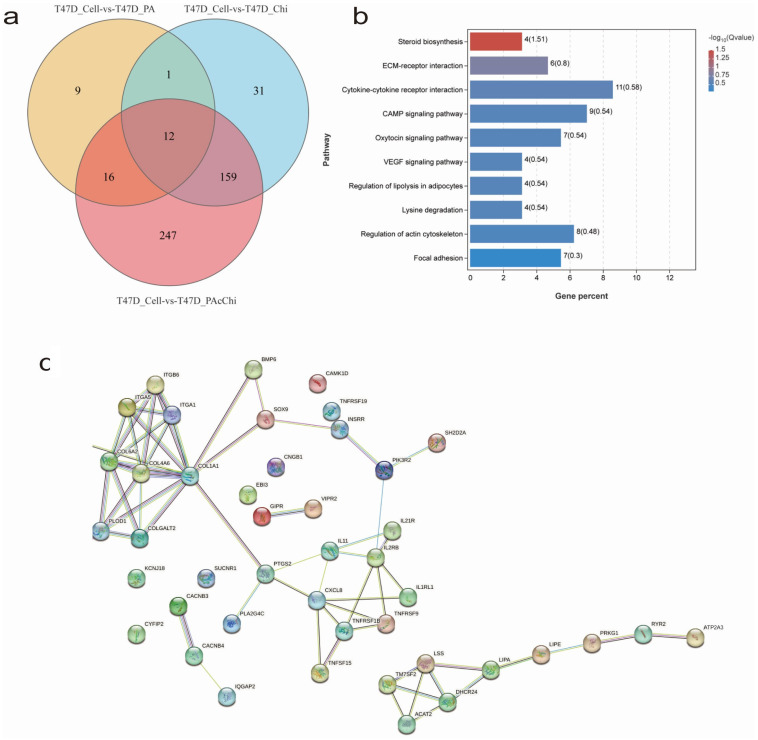
(**a**) Venn diagram illustrating the overlap of differentially expressed gene (DEG) in T47D cells treated with drugs. (**b**) Significantly associated pathways, up-regulated or down-regulated, possibly involved in the synergistic effect of proanthocyanidin (PA) and chidamide (Chi) on breast cancer cell characteristics or functions. (**c**) Protein–protein interaction (PPI) analysis of 48 DEGs related to T47D cells’ characteristics or functions using the STRING online tool.

To identify the pathways associated with the synergistic action of PA + Chi in breast cancer cells, transcriptome sequencing of twelve samples of T47D cell lines in four groups (control, PA, Chi, and PA + Chi) was performed. Genes showing a differential expression greater than 2-fold were identified using OmicShare tools. Genes that exhibited differential expression only in the PA or Chi treatment groups were excluded. A Wayne diagram was then constructed to illustrate the distribution of DEGs across the different groups, after excluding genes that were differentially expressed in the control group. As shown in the Wayne diagram, there were 38 DEGs in the PA group, 203 DEGs in the Chi group, and 436 DEGs in the PA + Chi group. After deducting the 187 genes found in the PA group and the Chi group, 247 genes remained in the PA + Chi group, potentially exerting a synergistic effect (Figure 3a).

To elucidate the pathways associated with the synergistic effect of PA + Chi in breast cancer, we performed a bioinformatic gene enrichment analysis of the 247 genes identified as potential candidates for synergy based on the transcriptome sequencing data. We also performed comprehensive pathway annotations, the widely used KEGG database. By analyzing pathways at the B-level classification, we identified 21 candidate pathways with a nominal *p* < 0.05 (Table 3). Further comparative analysis allowed us to identify the top 10 pathways, which encompassed 48 genes related to tumor function, growth, and development. These pathways included steroid biosynthesis pathway, extracellular matrix (ECM) receptor interaction pathway, cytokine–cytokine receptor interaction pathway, cAMP signaling pathway, oxytocin signaling pathway, VEGF signaling pathway, regulation of lipolysis in adipocytes pathway, lysine degradation pathway, regulation of actin cytoskeletal pathway, and adhesion plaque pathway (Figure 3b). To gain deeper insights into these pathways, protein–protein interaction (PPI) analysis was performed to examine the interplay between genes involved. The results indicated that the steroid biosynthesis pathway and ECM receptor interaction pathway were the most promising pathways associated with the synergistic effect of PA + Chi (Figure 3c).

## 3. Discussion

Breast cancer is a complex disease characterized by abnormal cell proliferation involving genetic and epigenetic changes. However, the clinical application of HDACi as a therapy for breast cancer is limited due to frequent toxicity and adverse effects, including those involving the blood, lymphatic, and gastrointestinal systems [26]. To overcome this challenge, the combination of a polyphenoic compound, PA, and HDACi, such as Chi, has been proposed as a strategy to enhance the therapeutic effect and reduce toxicity.

The findings of this study provide evidence, for the first time, that the combination of PA and Chi effectively inhibits breast cancer cells’ short-term and long-term proliferation, promotes apoptosis, and thus hold promise as a potential treatment for advanced breast cancer. Moreover, the synergistic effect observed in breast cancer cells was associated with the modulation of steroid biosynthesis and ECM receptor interaction pathways. 

The metabolic alterations observed in malignancies involve changes in lipid metabolism, and steroid biosynthesis plays a critical role in lipid production, with genetic alterations associated with hormone-related tumors, such as breast and prostate cancer [27,28,29]. In our study, we observed significant changes in the expression of genes that participated in steroid biosynthesis, including *LSS*, *DHCR24*, *TM7SF2*, and *LIPA*, after treatment with PA + Chi. Previous research has demonstrated the inhibitory effect of PAs on lipid metabolism and the expression of steroid metabolism-related enzymes, which can suppress breast cancer carcinogenesis [30,31,32]. Based on these findings, we hypothesize that the synergistic effect of PA + Chi on the steroid biosynthesis pathway contributes to their anti-tumor activity. Furthermore, we identified significant changes in genes related to ECM receptor interaction pathway, including *COL1A1*, *COL4A6*, *COL6A2*, *ITGA1*, *ITGA5*, and *ITGB6*, which are essential for cell support, signaling, invasion, and metastasis [33], suggesting that the modulation of the ECM receptor pathway may also contribute to the synergistic effect of PA + Chi on breast cancer cells.

Our study has certain limitations that need to be acknowledged. First, we did not confirm the entire pathway and specific genes involved in the observed synergistic effect. Additionally, it is important to consider the post-transcriptional regulation effect of HDACi, as it functions as an acetylation regulator. Further investigation employing techniques, such as short-hairpin RNA to interfere with specific gene expression and conducting in vivo experiments are warranted to validate the underlying mechanism of action of PA + Chi on steroid biosynthesis and ECM receptor pathways in breast cancer cells.

In conclusion, our results suggest that the combination of PA and the HDACi Chi may be a promising therapeutic approach for breast cancer. The mechanistic insights gained from our study provide valuable information for the development of novel strategies to treat advanced breast cancer, including drug-resistant forms of diseases.

## 4. Materials and Methods

### 4.1. Data Download, Processing, and Downstream Analysis

The raw data of single-cell RNA sequencing profile (GSE173634) were downloaded from the Gene Expression Omnibus (GEO) database. The count matrix data were processed using the Seurat package (version 4.1.1) of R software (version 4.1.3). A Seurat object was created and selected luminal cell types, including BT474, MCF7, T47D, and ZR751 for downstream analysis. The VlnPlot() function was used to investigate the expression levels of HDAC genes in each cell type.

### 4.2. Half Inhibitory Concentration Analysis (IC50) of Drugs Using the Cell Counting Kit 8 (CCK8)

The T47D cell line was obtained from our laboratory. The MDA-MB-231 cell line was obtained from Peking Union Medical College Cell Resource Center (Beijing, China). T47D and MDA-MB-231 cells were cultured in T25 flasks. Cells were then seeded into 96-well plates at a density of 5000–7000 cells/well, and cultured in logarithmic growth phase for 2 days. Once the cells adhered, 200 μL of 10% serum DMEM/1640 medium containing gradient concentrations of PA, Chi, or PA + Chi was added to each well, and the plates were incubated at 37 °C in a 5% CO_2_ incubator. After 72 h, 10% of CCK8 solution was added to each well, and the plates were incubated at 37 °C and 5% CO_2_ for approximately 2 h. The absorbance of each well at 450 nm was then measured using a microplate reader. Cell viability and IC50 values were calculated based on the absorbance readings.

### 4.3. Synergistic Effect Model Calculation and Short-Term Proliferation Analysis

The initial steps were similar to the cell IC50 analysis, wherein absorbance was measured and calculated as the cell inhibition rate. The obtained inhibition rates were then entered into the Compusyn1.0 software to determine the synergistic model.

For proliferation detection, cells in the logarithmic growth phase were seeded into 96-well plates at a density of 500–1000 cells/well and allowed to attach for 2 days. Subsequently, 10% serum DMEM medium containing a specific proportion of PA, Chi, or PA + Chi was added to each well, and the plates were cultured in an incubator at 37 °C and 5% CO_2_. The cell proliferation rate was calculated.

### 4.4. Long-Term Proliferation Analysis via Clone Formation

Cells in the logarithmic growth phase (500–1000) were seeded onto 6-well plates and incubated for 2 days until they attached to the surface. Then, the cells were treated with varying proportions of PA, Chi, or PA + Chi for approximately 2 weeks. After the formation of monoclonal colonies, the cells were fixed with 4% paraformaldehyde for 20 min and stained with 0.5% crystal violet for 15 min. At least 50 individual cells per monoclonal colony were counted in each well using ImageJ software (Version 1.8.0.172).

### 4.5. Cell Migration Analysis via Wound Healing Assay

Approximately 100 μL of cells was seeded from a T25 flask onto a 6-well plate. After 2 days of incubation, three horizontal and vertical lines were drawn to divide the wells. Then, 5% serum DMEM medium containing a specific proportion of PA, Chi, or PA + Chi was added and the plates were incubated. Samples were collected at 0 and 72 h for photography and analysis.

### 4.6. Cell Invasion Analysis Using Transwell Matrigel Invasion Assay

For cell invasion assays, approximately 10,000 T47D cells were seeded in 200 μL of no-serum medium onto the upper chamber of Transwell inserts coated with Matrigel. Then, 400 μL of 10% DMEM was added to the lower chamber of the Transwell. The chambers were incubated at 37 °C in a 5% CO2 incubator. After 48 h, the Transwell chambers were removed, and the cells were fixed and stained with 4% paraformaldehyde and 0.5% crystal violet, respectively. The number of invaded cells in five randomly selected fields of view for each chamber was counted and compared.

### 4.7. Cell Apoptosis and Intracellular Reactive Oxygen Species (ROS) Analysis via Flow Cytometry

The Annexin V-FITC/PI staining kit was used to detect cell apoptosis. For flow cytometry, the excitation light wavelength was set to 488 nm, FITC fluorescence was detected by a passband filter with a wavelength of 515 nm, and PI was detected by a filter with a wavelength greater than 560 nm. Cells in the right quadrant were compared.

To detect ROS levels, treated cells were incubated with a diluted 10 µM DHE fluorescent probe, allowing the samples to come into full contact with the probe. Flow cytometry was used to detect the fluorescence intensity at an excitation wavelength of 535 nm and emission wavelength of 610 nm. The P2 channel was used to delineate the ROS levels within the cells.

### 4.8. Gene Enrichment Analysis and KEGG Analysis of RNA Sequencing

Following the administration of treatments, transcriptome sequencing data were obtained from 12 samples with 3 replicates in 4 groups of T47D cells. The OmicShare tools (www.omicshare.com/tools) from Gene Denovo were used to identify differentially expressed genes (DEGs) in the treated groups compared to the control group, using a DEG threshold of FDR < 0.05 and FC ± 2. The DEGs were then grouped, and a Wayne diagram was drawn to select the synergistic-related DEGs. Common pathway annotation data were also used to identify significantly enriched signaling pathways.

### 4.9. Protein–Protein Interaction Analysis

The protein–protein interaction analysis was performed using the STRING online tool (version 11.5, https://cn.string-db.org/, accessed on 24 March 2023).

### 4.10. Statistical Analysis

Synergistic models with fractional effect (Fa) and CI values were analyzed using Compusyn (version 1.0), which is a simple computerized analytical simulation based on the median-effect principle of the mass-action law and its combination index theorem [34]. Statistical analyses in tumor growth and functions analysis part were conducted using GraphPad Prism9 software. Multi-comparisons between data were performed using one-way ANOVA (Brown–Forsythe and Welch ANOVA tests for data, which have non-equal standard deviations), and statistical significance was set at *p* < 0.05. Statistical analyses in KEGG pathways were previously mentioned. All statistical analyses in figures including *p*-value(s) were marked in figure legends.

## 5. Conclusions

In conclusion, our study demonstrates that the combination of PA and Chi exhibits a synergistic anti-tumor effect on hormone receptor-positive and triple-negative breast cancer cell lines. This combination effectively inhibited short-term and long-term tumor cell proliferation, while promoting apoptosis. These findings suggested that the mechanism underlying the synergistic effect of PA + Chi may be associated with these tumor phenotypes. Furthermore, our results indicate a potential involvement of the steroid biosynthesis pathway and ECM receptor interaction pathway in mediating the synergism observed with PA + Chi treatment.

## Figures and Tables

**Table 1:** The fractional effect and CI value of T47D cell after treatment with 18.5:1 ratio of proanthocyanidins and chidamide.

Combination (Drug A + Drug B)	PA Dose (μg/mL)	Chi Dose (μM)	Total Dose (PA + Chi)	Fractional Effect (Fa)	CI Value
Grape seed proanthocyanidin extract (GSPE/PA) + Chidamide (Chi)	27.75	1.5	29.25	0.433	0.75492
	37	2	39.00	0.552	0.70197
	46.25	2.5	48.75	0.705	0.53421
	55.5	3	58.50	0.773	0.49223
	64.75	3.5	68.25	0.806	0.49523
	74	4	78.00	0.783	0.62861

Combination index (CI) values were calculated using Compusyn (version 1.0). CI value < 1 indicates synergistic effect between drugs, and CI value > 1 indicates antagonistic effect between drugs.

**Table 2:** The fractional effect and CI value of MDA-MB-231 cells after treatment with 50:1 ratio of proanthocyanidins and chidamide.

Combination (Drug A + Drug B)	PA Dose (μg/mL)	Chi Dose (μM)	Total Dose (PA + Chi)	Fractional Effect (Fa)	CI Value
Grape seed proanthocyanidin extract (GSPE/PA) + Chidamide (Chi)	37.5	0.75	38.25	0.410	0.60335
	50	1	51.00	0.536	0.64555
	75	1.5	76.50	0.685	0.73750
	100	2	102.00	0.758	0.84109
	125	2.5	127.50	0.797	0.95470
	150	3	153.00	0.819	1.07840
	175	3.5	178.50	0.870	1.06536
	200	4	204.00	0.881	1.16653

Combination index (CI) values were calculated using Compusyn (version 1.0). CI value < 1 indicates synergistic effect between drugs, and CI value > 1 indicates antagonistic effect between drugs.

**Table 3:** Significant up-regulated or down-regulated pathways associated with synergistic PA + Chi synergistic effect in drug-treated breast cancer cell T47D.

Pathway(s)	Syn-DEGs (128)	* *p*-Value(s)
Arrhythmogenic right ventricular cardiomyopathy	7	0.00018945
Steroid biosynthesis	4	0.00025239
Hypertrophic cardiomyopathy	7	0.00051916
ECM-receptor interaction	6	0.00258582
Human papillomavirus infection	12	0.00626547
Cytokine–cytokine receptor interaction	11	0.00643997
cAMP signaling pathway	9	0.00842445
Oxytocin signaling pathway	7	0.01070732
Platelet activation	6	0.01383732
Dilated cardiomyopathy	7	0.014181
Small cell lung cancer	5	0.01420596
VEGF signaling pathway	4	0.01536765
Regulation of lipolysis in adipocytes	4	0.01536765
Mucin type O-glycan biosynthesis	3	0.01713699
Glycosaminoglycan biosynthesis—keratan sulfate	2	0.01862032
Lysine degradation	4	0.01888023
Protein digestion and absorption	5	0.02191461
Regulation of actin cytoskeleton	8	0.02415313
Bladder cancer	3	0.02580077
Focal adhesion	7	0.04042669
Cardiac muscle contraction	4	0.04997453

* All pathways mentioned are significantly different (* *p* < 0.05) in KEGG analysis; *p*-values are aligned in ascending order.

## Data Availability

The data presented in this study are available on request from the corresponding author. The data are not publicly available due to privacy issues.

## Supplementary Materials

The following supporting information can be downloaded at https://www.mdpi.com/article/10.3390/ijms241310476/s1.
