# Supplementary material for: The Synergistic Effect of Proanthocyanidin and HDAC Inhibitor Inhibit Breast Cancer Cell Growth and Promote Apoptosis
Source: Int J Mol Sci. 2023 Jun 22;24(13):10476. doi: 10.3390/ijms241310476 (PMC10341808; doi:10.3390/ijms241310476)
Supplement: Supplementary file 1 [file ijms-24-10476-s001.zip › ijms-2411558-supplementary.pdf]

Supplementary Figures
Figure S1. Cell viability curve and half inhibitory concentration (IC50) of proanthocyanidin (PA) and chidamide (Chi) in T47D and MDA-MB-231 cells
Figure S2. Proliferation differences on day 3 and day 5 after treatment

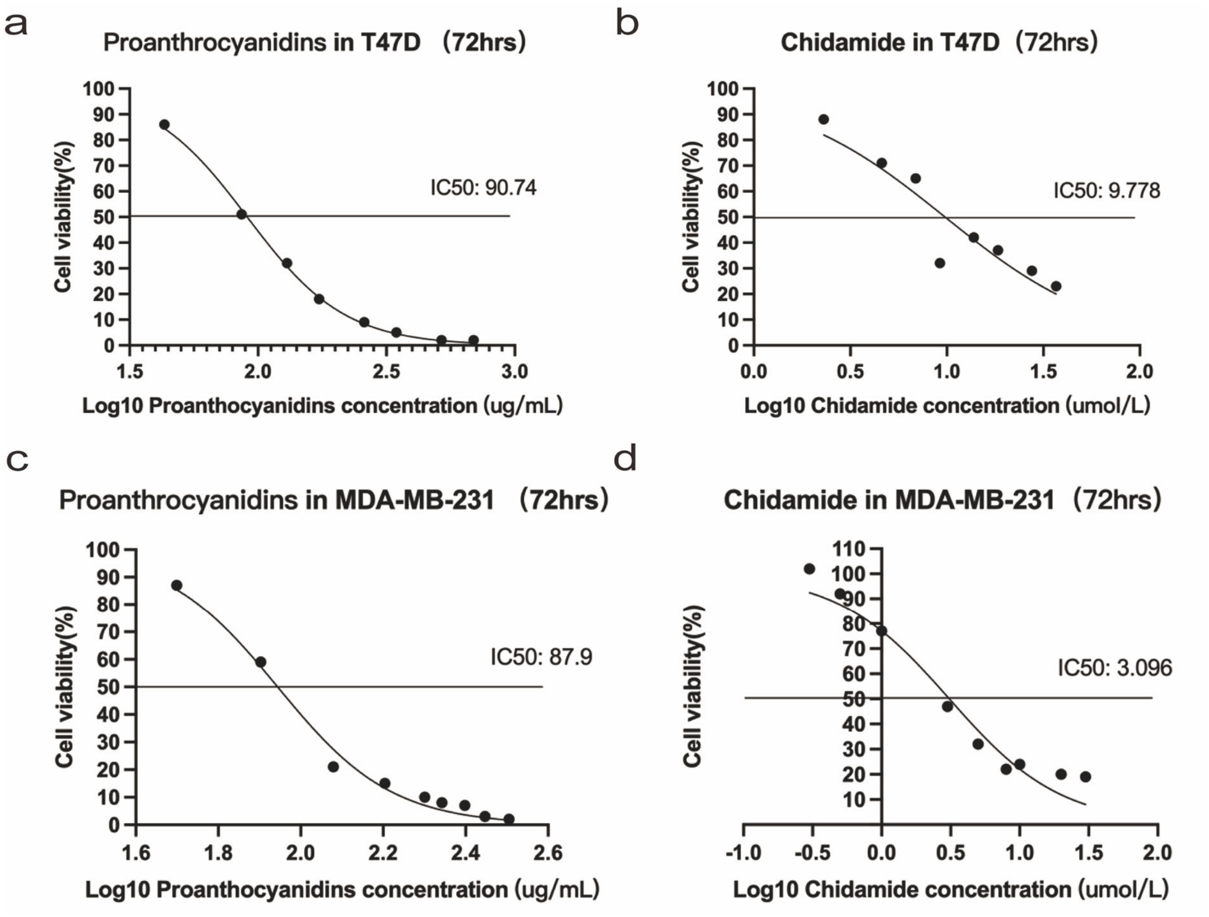

Figure S1
(a) Cell viability curve and half inhibitory concentration (IC50) of proanthocyanidins (PA) of T47D cell
(b) Cell viability curve and IC50 of chidamide (Chi) of T47D cell
(c) Cell viability curve and IC50 of PA of MDA-MB-231 cell
(d) Cell viability curve and IC50 of Chi of MDA-MB-231 cell

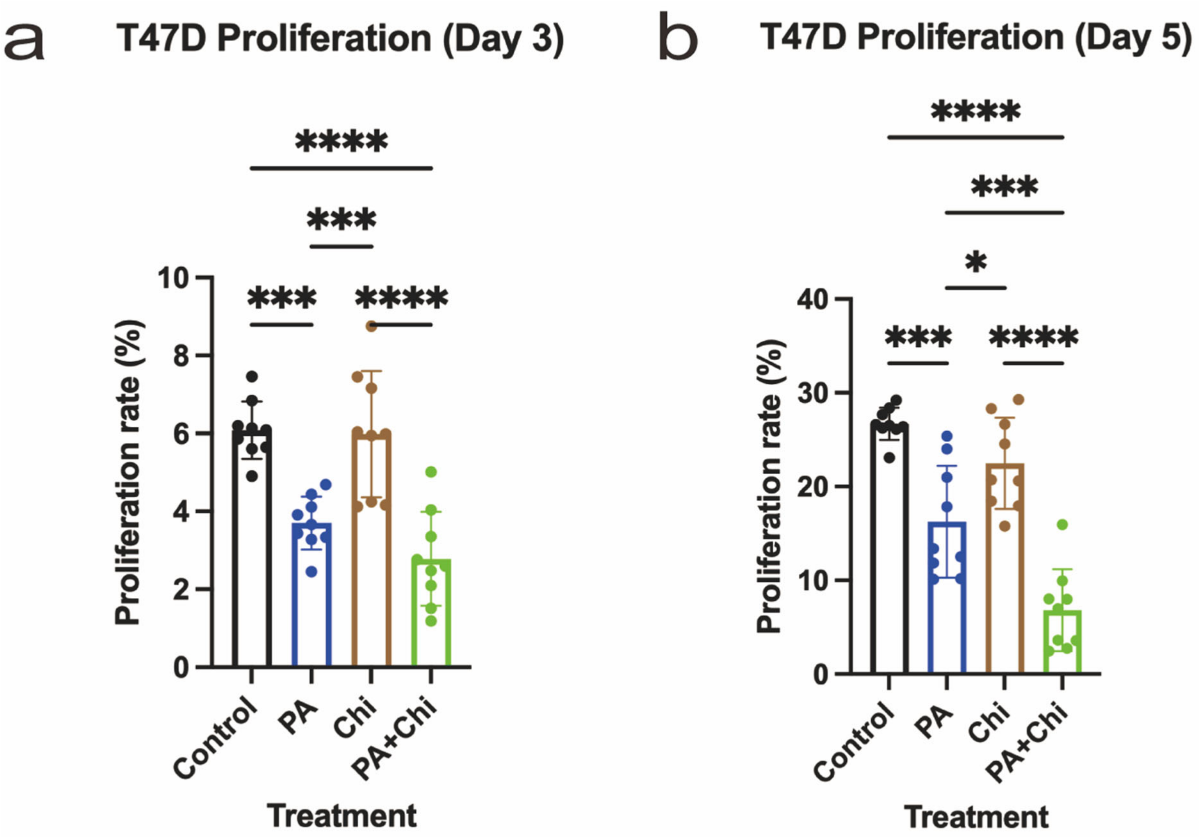

Figure S2

(a) The cell proliferation difference at day 3 after treatment. (For proliferation rate (%), n = 9 (Control), n = 9 (PA), n = 9 (Chi) and n = 9 (PA+Chi). ***$p<0.001$, or ****$p<0.0001$).

(b) The cell proliferation difference at day 5 after treatment. (For proliferation rate (%), n = 9 (Control), n = 9 (PA), n = 9 (Chi) and n = 9 (PA+Chi). *$p<0.05$, ***$p<0.001$ or ****$p<0.0001$).
